# When appendicitis mimics a liver abscess: diagnostic challenges in pediatric surgery

**DOI:** 10.1093/jscr/rjaf470

**Published:** 2025-09-20

**Authors:** Tomasz Waszak, Monika Wiczuk-Wiczewska, Adam Szymczak, Katarzyna Jończyk-Potoczna

**Affiliations:** Department of Pediatric Radiology, Institute of Pediatrics, Poznan University of Medical Sciences, Szpitalna 27/33, 60-572 Poznan, Poland; Student Scientific Society of Poznan University of Medical Sciences, Poznan, Poland; Student Scientific Society of Poznan University of Medical Sciences, Poznan, Poland; Department of Pediatric Radiology, Institute of Pediatrics, Poznan University of Medical Sciences, Szpitalna 27/33, 60-572 Poznan, Poland

**Keywords:** subhepatic abscess, acute appendicitis, pediatric surgery, hepatic abscess, abdominal imaging

## Abstract

Subhepatic abscesses are rare complications of acute appendicitis, often caused by delayed diagnosis and atypical presentation. We report the case of a 3-year-old male presenting with fever and abdominal pain, later diagnosed with a subhepatic abscess secondary to acute appendicitis. Abdominal imaging revealed multiple abscesses, including a primary subhepatic abscess adjacent to liver segments V and VI, and a retrocecal inflamed appendix with an appendicolith. The patient was treated with antibiotics and underwent laparotomy, drainage of the subhepatic abscess, and removal of a gangrenous appendix. Surgical exploration revealed inflammatory adhesions involving the duodenum and adjacent intestinal structures, which were meticulously dissected and managed. This case underscores the importance of high clinical suspicion and timely imaging in pediatric patients with atypical presentations of acute appendicitis.

## Introduction

Intra-abdominal abscesses are a rare but serious complication of acute appendicitis, occurring in 2%–6% of cases [1]. Among these, subhepatic abscesses represent an uncommon presentation, often associated with diagnostic and therapeutic challenges due to their atypical clinical and imaging features [1, 2]. The development of such abscesses typically results from delayed or incomplete treatment of appendicitis, leading to localized infection and abscess formation [1].

Pediatric patients with acute appendicitis are particularly at risk of complications due to their often non-specific symptoms, which can mask the underlying condition and delay diagnosis [3]. Subhepatic abscesses can present with right upper quadrant pain, fever, and signs of systemic inflammation, mimicking other intra-abdominal conditions such as hepatic abscesses or biliary pathology [1, 2]. Imaging studies, particularly contrast-enhanced computed tomography (CT), are essential for accurately identifying the source of infection and guiding appropriate management [4].

This case report describes a 3-year-old boy with a subhepatic abscess as a complication of acute appendicitis. Despite early empiric antibiotic therapy, imaging confirmed abscess formation requiring surgical intervention. Laparotomy revealed a large subhepatic abscess adjacent to the liver and duodenum, which was drained, and a gangrenous appendix was removed. The case emphasizes the importance of comprehensive diagnostic evaluation and timely treatment in preventing severe outcomes.

## Case report

A 3-year-old male was transferred by emergency medical services for evaluation of abdominal pain and fever. He had been hospitalized in a pediatric ward, where laboratory tests revealed significantly elevated inflammatory markers (CRP 226 mg/L, PCT 3.2 ng/ml). Two days later, repeat tests showed a slight decrease (CRP 190.8 mg/L, PCT 2.1 ng/ml). The patient’s medical history was unremarkable, and he was vaccinated according to the standard immunization schedule.

**Figure 1:**
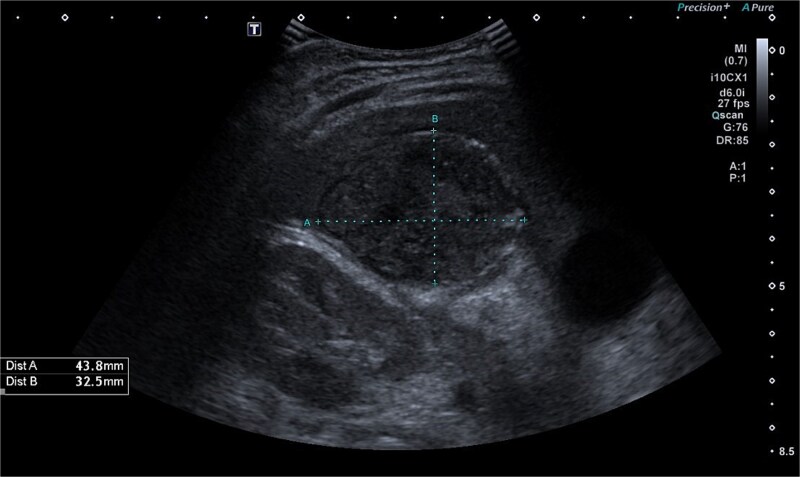
Abdominal ultrasonography imaging of pathological dense fluid collection adjacent to liver segment VI.

Abdominal ultrasonography identified a pathological, dense fluid collection in the right upper quadrant measuring 44 × 33 × 45 mm, located adjacent to segment VI of the liver (Fig. 1). The findings suggested an inflammatory lesion, possibly a hepatic abscess secondary to appendicitis with an atypically directed appendix. Empiric treatment with third-generation cephalosporins and metronidazole was initiated.

**Figure 2:**
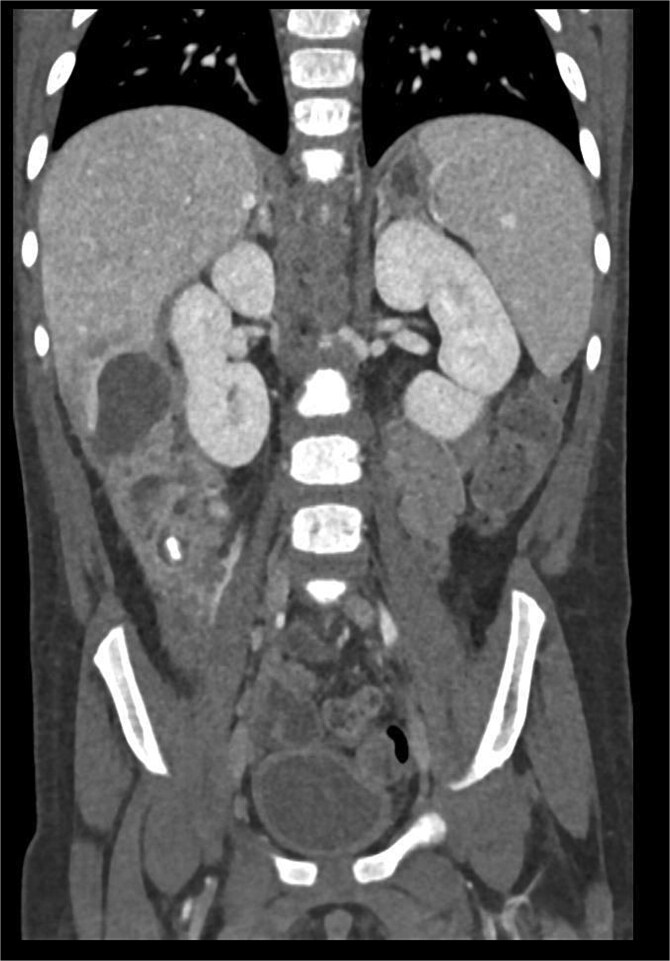
CT imaging of acute appendicitis with fecalith and multiple hepatic abscesses.

CT imaging of the abdomen (Fig. 2) revealed a retrocecal appendix in its typical location, measuring 50 mm in length and 14 mm in diameter, with a thickened wall (5.5 mm). A proximal fecalith measuring 3 × 3 × 14 mm was identified. Post-contrast enhancement of the appendix wall was noted. Adjacent to segments V and VI of the liver, a fluid collection with an air-fluid level was observed, measuring 5.3 × 3.8 × 4.5 cm, surrounded by a 3 mm enhancing capsule, consistent with a hepatic abscess. A second, smaller hypodense collection measuring 13 × 22 × 11 mm was found inferior to the primary abscess. Additional findings included reactive lymphadenopathy (up to 8 × 4 mm) and free fluid in the right iliac fossa (up to 12 mm). Other abdominal organs, including the liver, pancreas, kidneys, and spleen, appeared normal. The findings were consistent with complicated acute appendicitis and associated abscesses.

**Figure 3:**
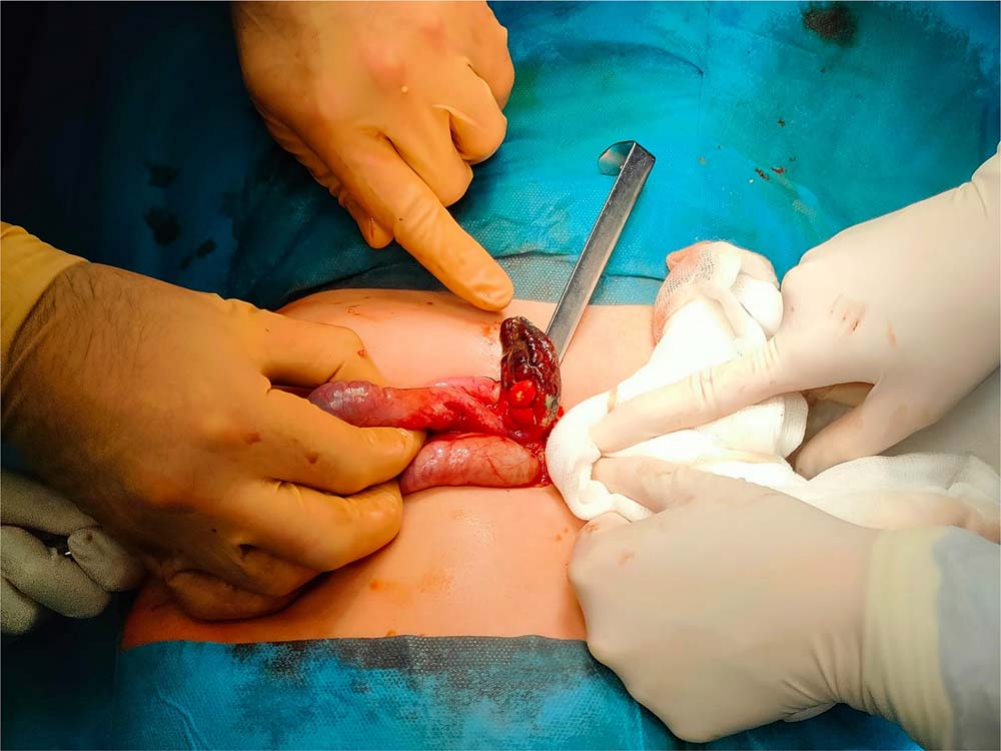
The gangrenous appendix revealed during surgery.

Surgical intervention was performed due to the patient’s clinical condition and imaging findings. Laparotomy revealed a large subhepatic abscess adjacent to the liver and duodenum. The abscess was drained, and surrounding areas were meticulously irrigated. Significant adhesions and inflammatory changes were noted involving the duodenum and nearby intestinal structures, which were carefully dissected. The appendix was completely gangrenous (Fig. 3) and was removed in its entirety. The appendiceal base was secured with two ligatures, and the surgical site was thoroughly irrigated before closing the abdominal wall. A drain was placed in the abscess cavity for postoperative management.

## Discussion

Subhepatic abscesses as a complication of acute appendicitis are rare but carry significant risks if not identified and treated promptly [2, 5]. The development of such abscesses often results from delayed diagnosis, incomplete treatment, or atypical presentations of appendicitis [3, 5]. In pediatric patients, the diagnosis of acute appendicitis can be particularly challenging due to non-specific symptoms such as vague abdominal pain, fever, or irritability, which can mask the severity of the condition [3, 4].

**Figure 4:**
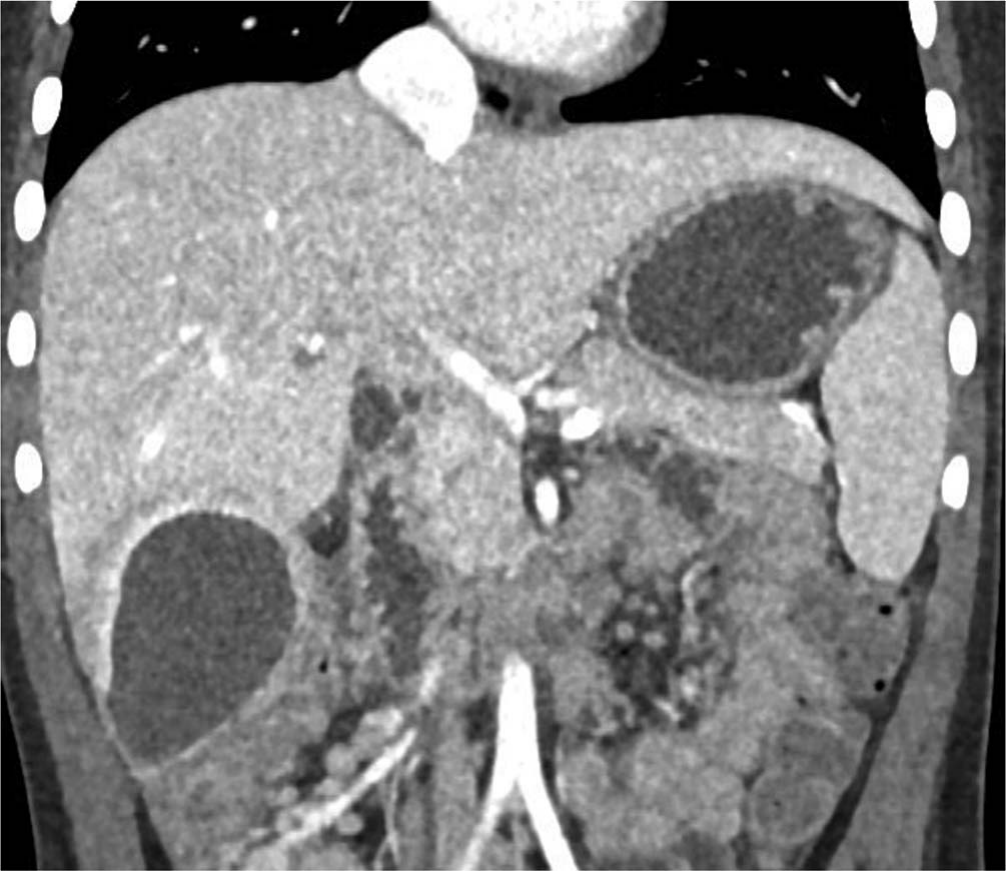
CT coronal view of a subhepatic abscess.

**Figure 5:**
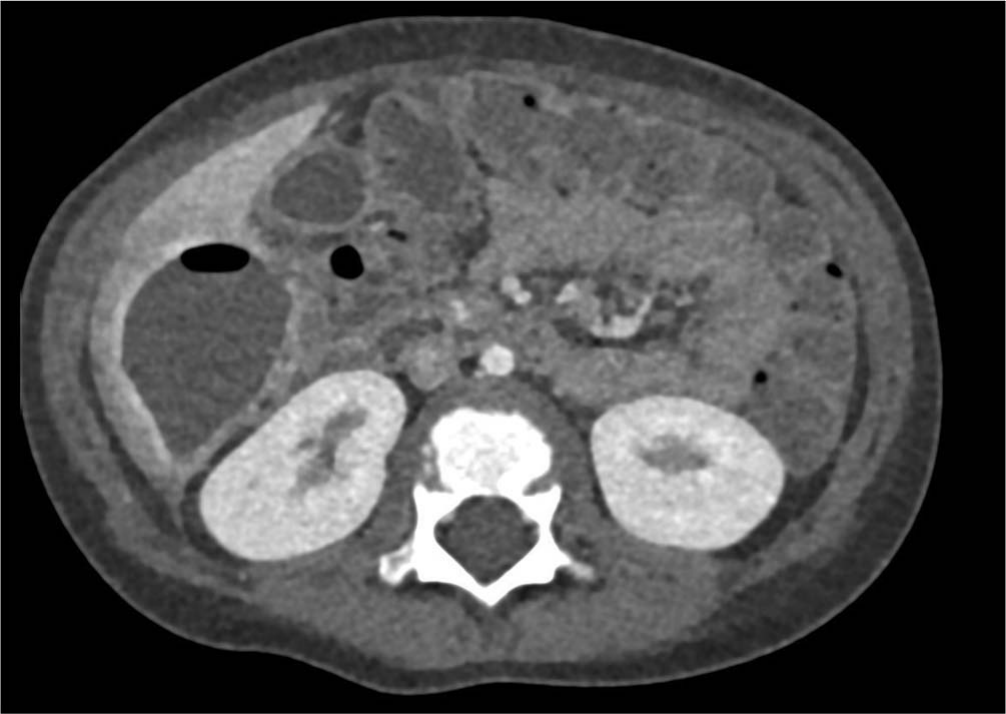
CT axial view of a subhepatic abscess. Contrast-enhanced capsule and fluid-air level visible.

Imaging plays a crucial role in diagnosing appendicitis and its complications. Ultrasound is often the initial modality used, but it may have limited sensitivity in detecting intra-abdominal abscesses, particularly when the appendix is not visualized or when findings are inconclusive [4, 5]. In such cases, contrast-enhanced CT is considered the gold standard, providing detailed visualization of the appendix and associated complications, including abscess formation [2, 4]. In the present case, the initial ultrasound findings suggested an atypical position of the appendix and a pathological fluid collection, but CT imaging confirmed a retrocecal appendix and subhepatic abscesses (Figs 4 and 5), guiding appropriate management.

The management of subhepatic abscesses involves a combination of antibiotics and surgical intervention. Empiric antibiotic therapy should target both aerobic and anaerobic organisms commonly associated with appendicitis, such as *Escherichia coli* and *Bacteroides fragilis* [2]. While percutaneous drainage is an option for selected patients, surgical drainage combined with appendectomy remains the definitive treatment for appendicitis-associated abscesses, particularly in cases with significant abscess formation or failed conservative management [6, 7].

In this case, surgical exploration revealed significant adhesions involving the duodenum and adjacent intestinal structures, necessitating careful dissection and management. Drainage of the abscess and removal of the gangrenous appendix were successfully performed, preventing further complications such as sepsis or generalized peritonitis [5, 6]. The patient’s recovery highlights the importance of multidisciplinary care, including pediatric surgeons and radiologists, in managing these complex cases.

## Conclusion

Subhepatic abscesses secondary to acute appendicitis are rare but significant complications that require prompt diagnosis and intervention. Anatomical variations in the position of the appendix must be considered in atypical cases of appendicitis. Advanced imaging modalities, particularly CT, are critical for accurate diagnosis and guiding management. In cases with extensive inflammatory changes and abscess formation, surgical intervention remains the mainstay of treatment, ensuring complete resolution of the infection. This case underscores the need for heightened clinical awareness and a multidisciplinary approach to optimize outcomes in pediatric patients with complex abdominal pathologies.
